# The Cincinnati Lancet and Observer

**Published:** 1858-02

**Authors:** 


### The Cincinnati Lancet and Observer.

We have received the first number of the above journal, being a combination of the *Western Lancet* and *Cincinnati Medical Observer*, to be edited by Drs. Mendenhall, Murphy, and Stevens. These journals have been among our most valued exchanges, and, with a consolidation of the *merits* of each, there will probably be no better journal in the country. We can confidently recommend it to those who would desire to have upon their tables one of the best representatives of Western Medicine.

The journal contains, monthly, sixty-four pages of reading matter, making a handsome volume of seven hundred and sixty-eight pages in the year, and furnished to subscribers at $3 per annum, with postage pre-paid to advance-paying subscribers; and is offered to Clubs of two or more, remitting at the same time, until the 1st of April, at $2.

